# Acute kidney injury in children: incidence, awareness and outcome—a retrospective cohort study

**DOI:** 10.1038/s41598-023-43098-7

**Published:** 2023-09-22

**Authors:** Flavia Chisavu, Mihai Gafencu, Ramona Stroescu, Alexandru Motofelea, Lazar Chisavu, Adalbert Schiller

**Affiliations:** 1 University of Medicine and Pharmacy ‘Victor Babes’, Timisoara, Romania; 2 Department of Paediatrics, ‘Louis Turcanu’ Emergency County Hospital for Children, rue Iosif Nemoianu, Number 2, 300041 Timisoara, Romania; 3 Centre for Molecular Research in Nephrology and Vascular Disease, Faculty of Medicine ‘Victor Babes’, Timisoara, Romania

**Keywords:** Diseases, Health care, Nephrology, Risk factors

## Abstract

The primary objective was to determine the epidemiologic influence of AKI awareness among physicians in a mixt paediatric population, including neonates. This single-centre, multiyear, observational retrospective study included all admitted patients between first of July 2014 and 31 December 2021. AKI was identified in 2194 patients out of the 128,036 hospital admissions with 129,936 serum creatinine measurements. Matching comparisons were used between AKI aware and AKI non-aware patients. The overall incidence of AKI was 1.65%. Stage 1 was identified in 24.24% of the AKI cases, stage 2 in 31.03% and stage 3 in 44.71%. The most prevalent cause of AKI was represented by prerenal AKI in 85.64% of the cases, followed by 12.16% renal causes respectively 2.18% postrenal causes. Exposure to sepsis, critical illness, hypovolemic shock and mechanical ventilation increased mortality by 2.09, 4.69, 4.64- and 4.93-times (*p* = 0.001). Cancer and heart failure increased mortality by 4.22 (*p* < 0.001) respectively 2.17 times (*p* = 0.001). The presence of AKI increased mortality by 79.11 times while only half of the AKI associated deaths were recognized by physicians. AKI increased hospitalization more than 4 times the average stay. AKI awareness was dependent of lower age and severity. Also, awareness increased mortality and prolonged hospitalization. 1 in 3 neonates and 1 in 4 children were AKI aware. The physician’s awareness of AKI diagnosis is in general low due to lack of appliance of current guidelines in exploring exposures and susceptibilities for AKI screening.

## Introduction

Acute kidney injury (AKI) is a growing global health burden. Worldwide, 1 in 5 adults and 1 in 3 children are experiencing AKI during a hospital admission^1^ with high comorbidity rate, high mortality and prolonged hospital stay^2^. Several large studies have drawn attention to the increasing number of AKI cases in the paediatric population mostly in the neonatal setting but also in critically ill patients and young adults^3–5^. Even though over 10 years have passed since a consensus definition of AKI was published, there is still a low awareness rate of AKI among physicians as the guidelines aren’t uniformly applied.

Current paediatric studies state the need for awareness in recognizing the risk of AKI and identifying susceptibilities and the need to correct different modifiable exposures for better outcomes^5–7^. The importance of adherence to current AKI guidelines represents the highlight of our study. The aim of our retrospective study was to identify the incidence of AKI, the awareness of AKI among physicians and factors that influenced awareness. In addition, we aimed to evaluate the impact of specific exposures and susceptibilities on the awareness of AKI and on AKI associated mortality and hospitalization.

## Material and methods

We conducted a multiyear, single-center, retrospective observational study in “Louis Turcanu” Emergency County Hospital for Children in Timisoara, Romania. Data were extracted from the electronic data base, between first of July 2014 and 31 December 2021. Out of the 128,036 admitted patients we identified 2194 children with AKI. One should mention that in 2020 and 2021, in Romania, during the COVID pandemic, nationwide measures have been imposed by the Ministry of Health, restricting hospital admittance while COVID cases were redirected to designated hospitals, As a result, the number of hospital admissions/year were 50% lower compared to previous years. All the methods were performed in accordance with the declaration of Helsinki. The experimental protocol was approved by “Louis Turcanu” Emergency County Hospital for Children from Romania Ethics Committee and is in accordance with Romanian legislation regarding the handling of human participants. The anonymity of the patients was maintained during the study protocol. In addition, at admittance in the hospital, the legal guardians of the patients signed an informed consent.

We classified the different age groups according to the Centre for Disease Control and Prevention (CDC) as: premature (all the babies born before 37 weeks of gestation), full-term new-borns, infants (between 28 days and 12 months of life), toddlers (over 12 months up to 3 years), pre-schoolers (between 3 and 5 years), scholars (6–11 years) and adolescents (12 to 18 years).

AKI was defined according to Kidney Disease Improving Global Outcomes (KDIGO) guidelines as an increase in serum creatinine by 26.5 µmol/l within 48 h or increase in serum creatinine up to 1.5 times baseline or over, known or presumed to have occurred within the prior 7 days for the hospitalized patients or the nadir serum creatinine in 7 days from admission^8^.

AKI staging was performed according to the KDIGO criteria based on creatinine level with an increase of more or equal to 26.5 µmol/l or increase to more than or equal to 150–200% (1.5- to twofold) from baseline in stage 1, more than 200–300% increase from baseline in stage 2 and > 300% increase from baseline or more than ≥ 354 µmol/l or the initiation of renal replacement therapy (RRT) in stage 3 AKI^8^ Stage 3 AKI was considered in all patients with documented anuria for over 12 h. Serum creatinine measurements were performed using the Abbott Jaffe method with plasma pediatric creatinine reference intervals based on age.

Kidney damage with duration between 7 and 90 days was defined as acute kidney disease (AKD)^8^.

Chronic kidney disease (CKD) was defined according to KDIGO CKD guideline as abnormalities of kidney structure or function (as evidenced by damage markers) for more than 3 months with health implications or glomerular filtration rate < 60 ml/min/1.73m^2^ for more than 3 months with or without damage markers^9^.

Data were analysed by two different operators in the same time in order to eliminate potential bias.

AKI awareness in different medical clinics was considered the recognition of AKI diagnosis at admission or at discharge according to the International Classification of Diseases—10th edition (ICD-10) Clinical Modification codes (N17.0, N17.1, N17.2, N17.8, N19, N99.0, and P96.0) or noted in the medical reports (expressions like “reduced renal function”, “elevation in serum creatinine”, “acute renal failure”). AKI non-aware was considered in patients presenting AKI according to KDIGO, without recognition in the medical records.

Following the recommendation of KDIGO AKI guideline and 2021 Consensus conference, we stratified the risk of AKI in both AKI aware and AKI non-aware groups through exposures and susceptibilities with direct impact on mortality and hospitalization. The considered exposures were: sepsis, critical illness, circulatory shock, trauma, major non-cardiac surgery, nephrotoxic drugs, poisonous plants and mechanical ventilation^8,10^. We excluded cardiac surgery, burns and radio contrast agents because the hospital doesn’t have a cardiac surgery department, major burns are redirected to a dedicated unit in the capital city of Romania and radio contrast induced AKI was difficult to demonstrate in the absence of follow-up in creatinine dynamics. We adjusted the susceptibilities of developing AKI in the paediatric population by including heart failure, arterial hypertension and stem cell transplant to the already stated: CKD, chronic diseases (heart, lung, liver), diabetes mellitus, cancer, anaemia, female gender and dehydration/volume depletion. We replaced advanced age with extreme age as prematurity represents an important susceptibility of AKI. We excluded black race since our electronic database didn’t include race.

Community acquired (CA) AKI was considered in all patients who presented AKI at admission. In hospital AKI (IH) was considered in all patients that developed AKI during hospitalization.

### Statistical analyses

Variables were analysed with the Chi-square test. All continuous variables were tested for normality using Shapiro–Wilk test. Data is presented as average ± standard deviation (SD), median and percentage for normally distributed continuous variables. For non-normally distributed continuous variables, the median and interquartile ranges (IQR) were reported, and groups were compared using the Wilcoxon Signed Ranks test. A log-rank test was conducted to determine the differences in the survival distribution in the three groups. Pairwise comparisons were conducted to determine which group had different survival distribution. The mortality risk was analysed using multivariate Cox proportional hazards models. In order to assess the independent factors that predict the risk of death in our cohort we employed a backward multivariate logistic regression model. An Akaike information criteria (AIC) was used in order to determine the best model. Odds ratio and 95% confidence interval (CI) were calculated. A linear ANOVA model was performed in order to determine the impact on hospitalizations. In this study, a *p*-value of 0.05 was considered the threshold for statistical significance. Data was analysed using SPSS v26 statistical software package.

## Results

**Figure 1:**
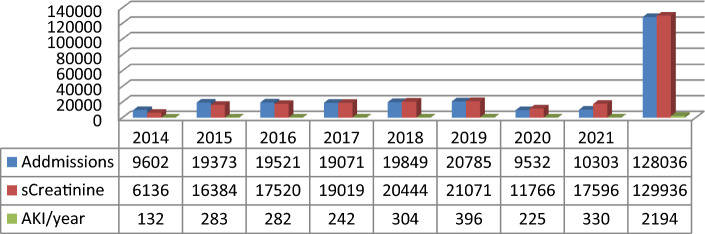
AKI episodes per year. *sCreatinine* serum creatinine level, *AKI* acute kidney injury.

Over the 8-years study period, we identified 2194 AKI patients out of the 128,036 hospital admissions with 129,936 serum creatinine measurements. The overall incidence of AKI was 17.11/1000 hospital admissions with a 2.33 times fold increased incidence over the 8 year period, from 13.74/1000 in 2014 to 32.02/1000 admissions in 2021 (Fig. 1).

Characteristics of AKI awareness and non-awareness on age, gender, demographics, stages, causes, AKD, CKD is shown in Table 1.

**Table 1:** Characteristics of AKI aware and non-aware on age, gender, demographics, IH, stages, causes, AKD and CKD.

AKI		Aware n = 591	Non-aware n = 1603	All patients n = 2194	*p* value
Age	Premature	161 (30.55)	366 (69.45)	527 (24.02)	0.032
	Full-term neonates	175 (31.3 )	384 (68.7)	559 (25.48)	0.007
	Infants	70 (29.41)	168 (70.59)	238 (10.84)	0.362
	Toddlers	26 (14.28)	156 (85.72)	182 (8.29)	< 0.001
	Pre-scholars	24 (16.43)	122 (83.57)	146 (6.65)	0.003
	Scholars	48 (20.68)	184 (79.32)	232 (10.57)	0.023
	Adolescents	88 (28.29)	223 (71.71)	311 (14.17)	0.560
Gender	Feminine	262 (27.99)	674 (72.01)	936 (42.66)	0.337
Environment	Urban	329 (26.85)	896 (73.15)	1225 (55.83)	0.924
IH-AKI		390 (29.06)	952 (70.94)	1342 (61.16)	0.005
AKI stages	1	80 (15.03)	452 (84.97)	532 (24.24)	< 0.001
	2	128 (18.79)	553 (81.21)	681 (31.03)	
	3	383 (39.04)	598 (60.96)	981 (44.71)	
AKI causes	Postrenal	28 (58.33)	20 (41.67)	48 (2.18)	< 0.001
	Prerenal	466 (24.8)	1413 (75.2)	1879 (85.64)	
	Renal	97 (36.32)	170 (63.68)	267 (12.16)	
AKD		209 (53.58)	181 (46.42)	390 (17.77)	< 0.001
CKD		23 (46.93)	26 (53.07)	49 (2.2)	< 0.001

*AKI* acute kidney injury, *IH* in hospital acquired AKI, *AKD* acute kidney disease, *CKD* chronic kidney disease, Values are expressed as n (%).

**Figure 2:**
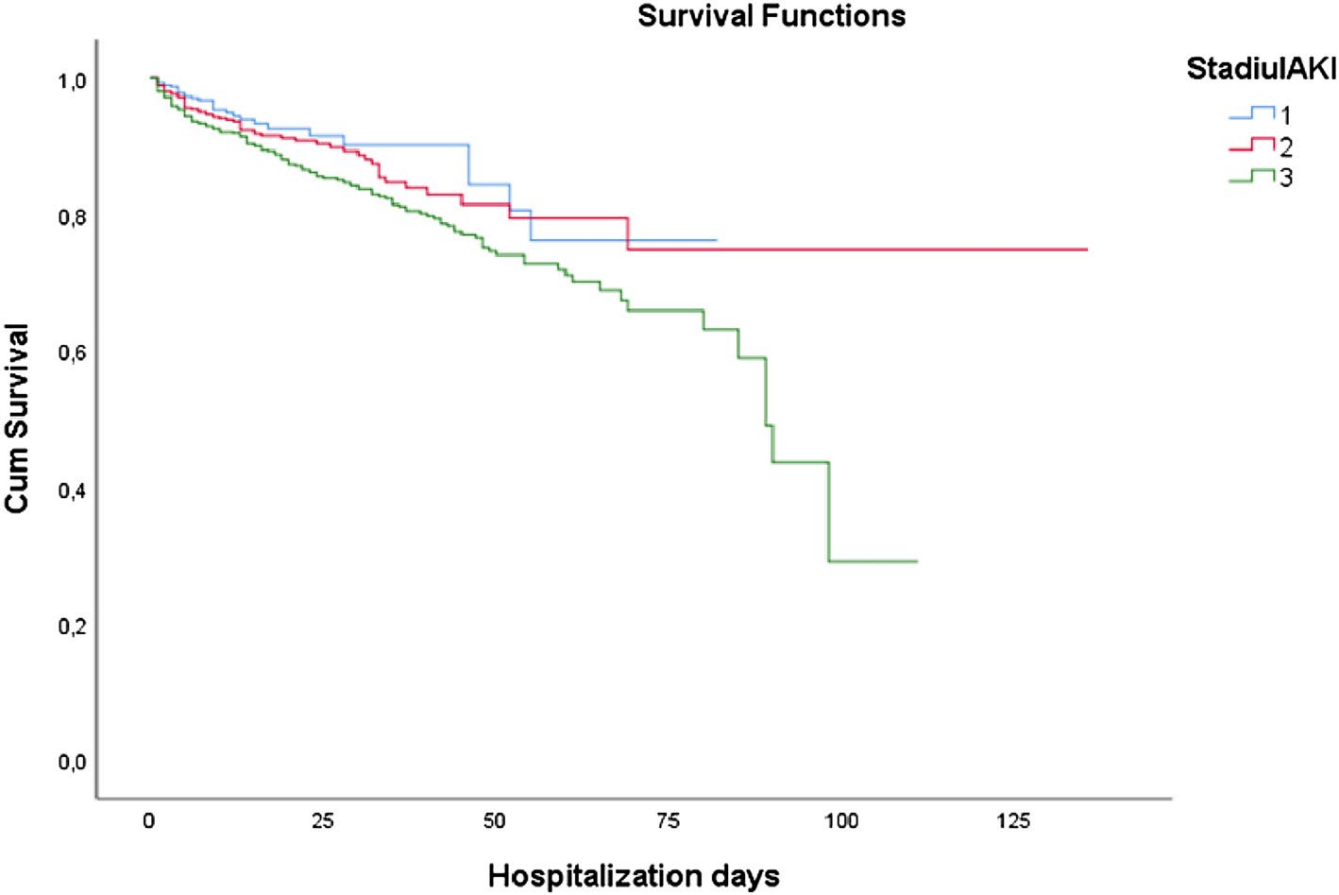
Kaplan–Meier survival curves in AKI stages and the impact on hospitalization days.

Out of the 128 036 admitted children 449 patients (0.35%) died of whom 255 (56.79%) presented AKI diagnosis. The risk of death in the presence of AKI was 79.11 times higher than in the no-AKI group (CI 65.9–94.8, *p* < 0.0001), mortality in the AKI group being 11.62%. Only stage 3 AKI group had a higher risk of death than stage 1 with an OR of 3.31 (CI 2.516–4.355, *p* < 0.001) Fig. 2.

The average hospitalization period for all admitted patients was 5.76 days. In the presence of AKI, aware and non-aware, the average hospitalization period increased to 20.9 days ± 19.3 days.

The overall awareness of AKI was 26.93%. AKI awareness was dependent of lower age being 30.55% in the preterm (*p* = 0.0321) and 31.3% in the full-term neonates (*p* = 0.007). We detected a slightly decreased awareness in the toddler age group 14.28%, (*p* < 0.001), in pre-scholars 16.43% (*p* = 0.003) and scholars 20.68% (*p* = 0.023).

AKI awareness was independent of gender or environment. We encountered a slightly lower awareness in CA-AKI (23.59%) as compared to IH-AKI (29.06%), *p* = 0.005.

Awareness increased with AKI stages, causes and the severity of kidney injury (*p* < 0.001). Stage 3 AKI had the highest awareness (39.04%) among doctors in contrast with stage 1 (15.01%) and stage 2 (18.79%) of the aware group. Regarding AKI causes we encountered the highest awareness rate in postrenal AKI (58.33%) followed by renal (36.32%) and prerenal AKI (24.8%). The awareness rates were 53.58% in AKD (*p* < 0.001) respectively 46.93% in CKD patients (*p* = 0.001).

We performed risk assessment of developing AKI on the whole cohort to see if exposed to different known renal insults or the presence of personal susceptibilities increases AKI recognition among specialists Table 2. Sepsis (52.59%), mechanical ventilation (43.66%) and nephrotoxins (22.1%) were the most prevalent exposures. Once the patients were exposed to sepsis (*p* < 0.001), critical illness (*p* < 0.001), hypovolemic shock (*p* < 0.001), trauma (*p* = 0.002) and mechanical ventilation (*p* < 0.001) the awareness increased. Dehydration or volume depletion (96.8%) and anaemia (69.69%) were the most prevalent susceptibilities. Volume depletion/dehydration (*p* = 0.031), CKD (*p* = 0.034), cancer (*p* = 0.055), anaemia (*p* = 0.004), heart failure (*p* < 0.001) and prematurity (*p* = 0.033) increased awareness.

**Table 2:** AKI awareness in exposures and susceptibilities.

AKI		Aware n = 591	Non-aware n = 1603	All patients n = 2194	*p* value
Exposure	Mechanical ventilation	341 (57.7)	617 (38.5)	958 (43.66)	< 0.001
	Sepsis	397 (67.2)	757 (47.2)	1154 (52.59)	< 0.001
	Clinical illness	106 (17.9)	100 (6.2)	206 (9.38)	< 0.001
	Hypovolemic shock	32 (5.4)	38 (2.4)	70 (3.19)	< 0.001
	Trauma	4 (0.7)	46 (2.9)	50 (2.27)	0.002
	Major non-cardiac surgery	62 (10.5)	179 (11.2)	241 (10.98)	0.653
	Nephrotoxins	121 (20.5)	364 (22.7)	485 (22.1)	0.527
	Poisonous plants	62 (10.5)	193 (12)	255 (11.62)	0.315
Susceptibility	Dehydration/Volume depletion	580 (98.1)	1544 (96.3)	2124 (96.8)	0.031
	Chronic kidney disease	17 (2.9)	24 (1.5)	41 (1.86)	0.034
	Chronic disease (heart, liver and lung)	44 (7.4)	127 (7.9)	171 (7.79)	0.711
	Diabetes mellitus	52 (8.8)	146 (9.1)	198 (9.02)	0.823
	Cancer	32 (5.4)	125 (7.8)	157 (7.15)	0.055
	Anaemia	439 (74.3)	1090 (68)	1529 (69.69)	0.004
	Prematurity	89 (15.1)	187 (11.7)	276 (12.57)	0.033
	Heart failure	73 (12.4)	90 (5.6)	163 (7.42)	< 0.001
	Arterial hypertension	28 (4.7)	51 (3.2)	79 (3.6)	0.083
	Stem cell transplant	2 (0.3)	3 (0.2)	5 (0.22)	0.51
	Female gender	262 (44.3)	674 (42)	936 (42.66)	0.337

*AKI* acute kidney injury, *n* number of patients, Values are expressed as n (%).

Exposed to known renal insults along with the personal susceptibilities of developing AKI resulted in increased AKI severity (stages 2 and 3) as well as a predominance of prerenal injury as seen in Table 3.

**Table 3:** Risk assessment of exposure and susceptibility on developing AKI based on stages and causes.

AKI		Stage 1 n = 532	Stage 2 n = 681	Stage 3 n = 981	p value	Prerenal n = 1879	Renal n = 267	Postrenal n = 48	Total n = 2194	*p* value
Exposure	Mechanical ventilation	136 (14.19)	267 (27.87)	555 (57.94)	< 0.001	891 (93)	55 (5.74)	12 (1.26)	958 (43.7)	< 0.001
	Sepsis	162 (14.03)	324 (28.07)	668 (57.9)	< 0.001	1061 (91.94)	87 (7.53)	6 (0.53)	1154 (52.6)	< 0.001
	Critical illness	21 (10.19)	50 (24.27)	135 (65.54)	< 0.001	182 (88.34)	22 (10.67)	2.0 (0.99)	206 (9.4)	0.342
	Hypovolemic shock	13 (18.57)	14 (20)	43 (61.43)	.016	61 (87.13)	9 (12.87)	0 (0)	70 (3.2)	0.443
	Trauma	27 (54)	13 (26)	10 (20)	< 0.001	42 (84)	7 (14)	1 (2)	50 (2.3)	0.921
	Major non-cardiac surgery	49 (20.33)	76 (31.53)	116 (48.14)	.295	216 (89.62)	15 (6.22)	10 (4.16)	241 (11)	0.001
	Nephrotoxins	116 (23.91)	158 (32.57)	211 (43.52)	.328	343 (70.72)	129 (26.59)	13 (2.69)	485 (22.1)	< 0.001
	Poisounous plants	68 (26.66)	84 (32.94)	103 (40.4)	.327	137 (53.72)	115 (45.09)	3 (1.19)	255 (11.6)	< 0.001
Susceptibility	Dehydration/volume depletion	499 (23.49)	660 (31.07)	965 (45.44)	< 0.001	1834 (86.34)	249 (11.72)	41 (1.94)	2124 (96.8)	< 0.001
	Chronic kidney disease	23 (56.09)	8 (19.51)	10 (24.4)	< 0.001	19 (46.34)	17 (41.46)	5 (12.2)	41 (1.9)	< 0.001
	Chronic diseases (leart, lung, liver)	34 (19.88)	63 (36.84)	74 (43.28)	.169	152 (88.88)	16 (9.35)	3 (1.77)	171 (7.8)	0.451
	Diabetes mellitus	44 (22.22)	74 (37.37)	80 (40.41)	.13	178 (89.89)	17 (8.58)	3 (1.53)	198 (9)	0.201
	Cancer	51 (32.48)	53 (33.75)	53 (33.77)	.008	63 (40.12)	91 (57.96)	3 (1.92)	157 (7.2)	< 0.001
	Anemia	289 (18.9)	451 (24.49)	789 (56.61)	< 0.001	1321 (86.39)	186 (12.16)	22 (1.45)	1529 (69.7)	0.001
	Prematurity	26 (9.42)	73 (26.44)	177 (64.14)	< 0.001	268 (97.1)	8 (2.9)	0 (0)	276 (12.6)	< 0.001
	Heart failure	25 (15.33)	43 (26.38)	95 (58.29)	< 0.001	143 (87.73)	19 (11.65)	1 (0.62)	163 (7.4)	0.346
	Arterial hypertension	26 (32.91)	18 (22.78)	35 (44.31)	.114	35 (44.3)	41 (51.89)	3 (3.81)	79 (3.6)	< 0.001
	Stem cell transplant	2 (40)	1 (20)	2 (40)	.693	3 (60)	2 (40)	0 (0)	5 (0.22)	0.159
	Female gender	227 (24.25)	291 (31.08)	418 (44.67)	.999	807 (86.21)	115 (12.28)	14.0 (1.51)	936 (42.7)	0.161

*AKI* acute kidney injury; *n* number of patients, Values are expressed as n (%).

**Figure 3:**
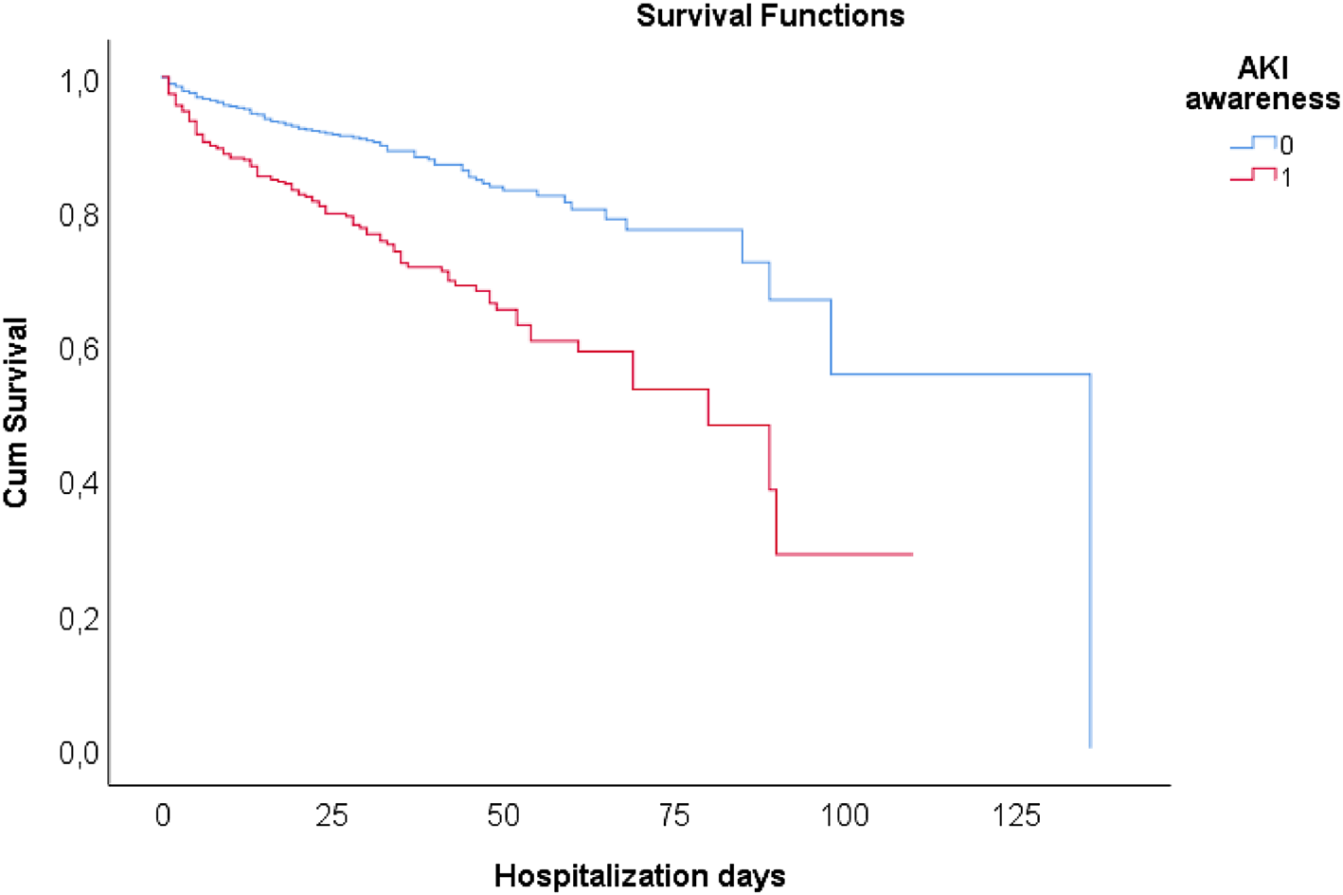
Kaplan–Meier survival curves of the AKI groups and the impact on hospitalization days. *AKI* acute kidney injury, *0* acute kidney injury non-aware; *1* acute kidney injury aware, *0* AKI non-aware (blue line), *1* AKI aware (red line).

In the deceased patients with associated AKI, the awareness was 50.68% (129 out of 255 patients). Being aware of AKI increased the mortality by 1.39-times (CI 1.03–1.88, *p* = 0.033) Fig. 3.

Exposure to sepsis, critical illness, hypovolemic shock and the need for mechanical ventilation increased mortality by 2.09 (CI 1.4–3.12), 4.69 (CI 3.21–6.84), 4.64- (CI 2.56–8.44) and 4.93-times (CI 3.23–7.52), *p* < 0.001. Cancer and heart failure were the only susceptibilities encountered that statistically increased mortality by 4.22 (CI 2.1–8.48, *p* < 0.001) and 2.17 times (CI 1.35–3.48, *p* = 0.001) respectively (Supplementary Table 1).

Non-aware AKI had an average of 20.1 ± 19.1 days hospital stay while AKI awareness increased the hospital length to 22.8 days ± 19.7 days, *p* = 0.004.

## Discussions

To our knowledge, this study represents the largest epidemiological assessment of AKI awareness in the paediatric population from Europe. The incidence of AKI in our cohort (1.65%) was lower than previously reported^1,2,11–14^ but still higher than in the most extensive multicentre AKI study from United States (0.4%)^15^. We justify these results by the reduced number of serum creatinine measurements as seen in Fig. 1. The latest paediatric meta-analysis of AKI reports a higher AKI incidence in children, 26%, excluding neonates^16^.

AKI awareness represents a global health problem with substantially underdiagnosed patients as previously documented in a large study on hospitalized children in China where up to 96% of the AKI events not being diagnosed on the discharge records^4^. Though the ICD-10 coding system is known to underestimate the AKI diagnosis^17^, our 26.93% overall awareness of AKI was similar to the multicentre study on the incidence of AKI in children hospitals across England where recognition and management of AKI was seen in just over 25% of the children^18^. Previous published data had shown that using a care bundle can improve the recognition of AKI^19,20^.

The largest age group in our study was represented by children below 1 month of life (49.5%) with a much higher incidence of AKI than the one reported by Sutherland (19%)^15^. Albeit the neonatal setting showed the highest awareness in our cohort, 3–12 years old children were most likely AKI misdiagnosed by physicians.

In our study, CA-AKI represented 38.8% of the admitted patients, similar to others^4^. with a slightly increased awareness rate for the IH acquired AKI (29.06%).

The severity of AKI and the extension in time of increased serum creatinine is associated with worse outcome as previously published in AWAKEN^3^, AWARE^4^, Baby-Ninja^7^, AKI-EPI^21^ studies as well as in many other regional studies in both critically and non-critically ill patients^1,13,22,23^. We report a high incidence of AKD in children (17.8%) with half of these patients being reported as AKI.

Prerenal states were the main cause of AKI in our cohort. AKI severity was correlated to the underlying disease. Exposure to mechanical ventilation, sepsis, critically illness, hypovolemic shock and trauma increased AKI awareness. The susceptible children with chronic diseases (heart failure, CKD, cancer), preterm babies, and hypovolemia increased AKI awareness among physicians.

AKI has evolved from a primary single renal disease to a syndrome secondary to other systemic illnesses or its treatment^24^ with high mortality. Awareness of AKI is mandatory in order to identify the cause of renal injury especially for CA-AKI using an algorithmically approach. Even though the overall mortality rate was low, the presence of AKI (aware and non-aware) increased the mortality risk by 79.11 times, significantly higher than the 4.6 AKI related mortality risk reported by Meena in the recent AKI meta-analysis^16^. The pooled mortality in the AKI pediatric group reported in the 2012 meta-analysis of AKI by Susantitaphong^1^ was 13.8%, similar to our results (11.6%), as well as the 2023 AKI meta-analysis (11%)^16^. As a result of poor AKI screening among hospitalized patients, with reduced serum creatinine follow-up and the absence of urinary output measurement, the real incidence of AKI in our cohort is most likely higher than reported thus the relative risk of death decreases.Awareness of AKI increased mortality by 1.39 times as a result of AKI severity.

Exposure to sepsis, critical illness, hypovolemic shock and the need for mechanical ventilation increased the mortality significantly, similar to the previously published results^23,25^, while the most significant susceptibilities encountered with impact on mortality were cancer and heart failure..

AKI is associated with prolonged hospitalization and higher costs. In our study, hospital stay among AKI patients was almost 4 times longer than in non-AKI children. In the aware group of AKI, hospitalization increased compared to non-aware AKI as a result of AKI severity. One should note that a patient often presents with a handful of susceptibilities over which, if not aware of them, will be exposed to renal insults.

In the 2016 Epidemiology of acute kidney injury in children worldwide, including developing countries^26^, Lameire emphasized the dramatic rise in the incidence of AKI and the imperative need for future studies addressing the true incidence and outcomes of AKI as well as an increase in AKI awareness among primary caregivers who have insufficient awareness of the diagnosis and its management, thus failing to rapidly implement the simple, inexpensive measures that would have the highest beneficial impact. Truly this is the end-point of our study as well. More than 10 years have passed since AKI—KDIGO guideline was published and AKI is still underdiagnosed. A raising global concern regarding AKI has led to the Consensus-Based Recommendations on Priority Activities to Address Acute Kidney Injury In Children in 2022 that emphasize the need of AKI awareness, proper management and long term follow-up ^27^

In our study, 1 in 3 neonates and 1 in 4 children were AKI aware. Considering AKI as a piece of puzzle, the treatment of the underlying disease should follow a logical approach with careful consideration of secondary kidney injury in a susceptible individual.

We are aware that our study has several limitations, being single-centre, retrospective and observational. Without follow-up after discharge. Another limitation was the lack of urine output measurement for all patients. The strong points are the high number of patients, including neonates with a wide spectrum of diseases, a retrospective image of AKI awareness among physicians and the associated outcomes during hospitalization.

### Supplementary Information

Supplementary Table 1.

## Data Availability

The data underlying this article will be shared on reasonable request to the corresponding author.
