# Supplementary material for: Acute kidney injury in children: incidence, awareness and outcome—a retrospective cohort study
Source: Sci Rep. 2023 Sep 22;13:15778. doi: 10.1038/s41598-023-43098-7 (PMC10516941; doi:10.1038/s41598-023-43098-7)
Supplement: Supplementary file 1 — Supplementary Table 1. [file 41598_2023_43098_MOESM1_ESM.docx]

**Supplemental Table 1. Model coefficients of death on risk assessment of AKI aware versus AKI non-aware**

|  |  | | **95% Confidence Interval** | |  | | | | **95% Confidence Interval** | |
| --- | --- | --- | --- | --- | --- | --- | --- | --- | --- | --- |
|  | **Predictor** | **Estimate** | **Lower** | **Upper** | **SE** | **Z** | **p** | **Odds ratio** | **Lower** | **Upper** |
| **Exposure** | Mechanical ventilation | 1.5957 | 1.1725 | 2.019 | 0.216 | 7.3906 | < .001 | 4.93167 | 3.23008 | 7.5296 |
|  | Sepsis | 0.7387 | 0.3371 | 1.14 | 0.205 | 3.6054 | < .001 | 2.09311 | 1.40089 | 3.1274 |
|  | Critical illness | 1.5461 | 1.1687 | 1.923 | 0.193 | 8.0296 | < .001 | 4.69302 | 3.21778 | 6.8446 |
|  | Hypovolemic shock | 1.5369 | 0.9406 | 2.133 | 0.304 | 5.0517 | < .001 | 4.64999 | 2.56148 | 8.4414 |
|  | Trauma | -1.3738 | -3.5012 | 0.754 | 1.085 | -1.2657 | .206 | 0.25314 | 0.03016 | 2.1245 |
|  | Major non-cardiac surgery | -14.0503 | -850.1332 | 822.033 | 426.581 | -0.0329 | .974 | 7.91E-07 | 0 | Inf |
|  | Nephrotoxins | 13.0497 | -823.0333 | 849.133 | 426.581 | 0.0306 | .976 | 464963.7 | 0 | Inf |
|  | Poisonous plants | -13.5011 | -849.584 | 822.582 | 426.581 | -0.0316 | .975 | 1.37E-06 | 0 | Inf |
| **Susceptibility** | Dehydration/volume depletion | 0.4892 | -0.9792 | 1.958 | 0.749 | 0.653 | .514 | 1.63105 | 0.3756 | 7.0828 |
|  | CKD | 0.9176 | -0.3203 | 2.155 | 0.632 | 1.4529 | .146 | 2.50316 | 0.72596 | 8.6311 |
|  | Chronic disease: heart, liver, lung | 0.6328 | -1.1308 | 2.396 | 0.9 | 0.7032 | .482 | 1.88279 | 0.32277 | 10.9828 |
|  | Diabetes mellitus | -0.0708 | -1.7956 | 1.654 | 0.88 | -0.0804 | .936 | 0.93167 | 0.16604 | 5.2278 |
|  | Cancer | 1.4406 | 0.7423 | 2.139 | 0.356 | 4.0434 | < .001 | 4.22308 | 2.10073 | 8.4896 |
|  | Anemia | 0.2952 | -0.1731 | 0.763 | 0.239 | 1.2354 | .217 | 1.34334 | 0.84104 | 2.1456 |
|  | Prematurity | 0.0456 | -0.3649 | 0.456 | 0.209 | 0.2179 | .828 | 1.0467 | 0.69429 | 1.578 |
|  | Heart failure | 0.7749 | 0.3017 | 1.248 | 0.241 | 3.21 | .001 | 2.17029 | 1.35222 | 3.4833 |
|  | Arterial hypertension | -1.247 | -2.2879 | -0.206 | 0.531 | -2.3479 | .019 | 0.28738 | 0.10148 | 0.8138 |
|  | Stem cell transplant | -11.2486 | -1249.44 | 1226.942 | 631.742 | -0.0178 | .986 | 1.30E-05 | 0 | Inf |
|  | Female gender | 0.2612 | -0.0457 | 0.568 | 0.157 | 1.6682 | .095 | 1.29842 | 0.95535 | 1.7647 |

AKI=acute kidney injury; CKD=chronic kidney disease; Estimates=log odds of death between AKI aware and AKI non-aware;
